# Charge-Balanced Design for Redox-Responsive Disassembly of Ampholytic β-Sheet Peptide Nanofibers

**DOI:** 10.3390/polym18111291

**Published:** 2026-05-24

**Authors:** Tomonori Waku, Kaede Akita, Takehiro Deromachi, Kazuya Matsuo, Akio Kobori

**Affiliations:** 1 Faculty of Molecular Chemistry and Engineering, Kyoto Institute of Technology, Matsugasaki, Sakyo-ku, Kyoto 606-8585, Japankmatsuo@kit.ac.jp (K.M.); akobori@kit.ac.jp (A.K.); 2 Center for Social and Biomedical Engineering, Kyoto Institute of Technology, Matsugasaki, Sakyo-ku, Kyoto 606-8585, Japan

**Keywords:** redox responsiveness, stimuli-responsive disassembly, β-sheet peptide nanofibers, self-assembling peptides, drug delivery

## Abstract

Self-assembling β-sheet-forming peptides are attractive building blocks for drug delivery nanomaterials. However, their strong intermolecular interactions often lead to high structural stability, which can hinder intracellular dissociation and limit cargo availability. Here, we propose a charge-compensated ampholytic design strategy for β-sheet peptide nanofibers that undergo destabilization and disassembly under reducing conditions. Six ampholytic peptides comprising an anionic main-chain peptide (β-sheet-forming motif, model antigenic cargo, and oligoglutamic acid segment) and a disulfide-linked cationic segment were designed and synthesized to vary the lengths and charges of the anionic and cationic segments, as well as the cationic insertion position. Four peptides formed nanofibers in 4×phosphate buffered saline (4×PBS) and the resulting nanofibers remained stable after dilution to 1×PBS, retaining β-sheet-rich secondary structures and fibrillar morphologies for at least 24 h. Under reducing conditions, the four preformed nanofibers exhibited distinct behaviors, including reduction-insensitive persistence, disassembly, and transient destabilization followed by re-stabilization, depending on peptide charge design. Redox-triggered disassembly was favored when the main-chain peptide had sufficient anionic character and the cationic segment was of moderate length and charge. This study therefore provides a molecular design strategy for controlling the destabilization of β-sheet peptide nanofibers under reducing conditions through disulfide-cleavage-induced disruption of charge compensation.

## 1. Introduction

Self-assembling β-sheet-forming peptides have been widely studied as building blocks for functional materials because they can form ordered higher-order assemblies, such as nanofibers, in aqueous environments [1,2]. In particular, for drug delivery system (DDS) applications, covalent conjugation of cargo molecules, including anticancer drugs, antigenic peptides, and therapeutic peptides, to β-sheet-forming peptides can enable the straightforward fabrication of nanofibers with high cargo loading [3,4]. Furthermore, additional functional motifs, such as targeting ligands, can be incorporated into these assemblies through modular co-assembly, in which separately designed components are mixed to form a single assembly [5]. Such modularity can simplify formulation design and improve manufacturing reproducibility. On the other hand, β-sheet assemblies can exhibit strong intermolecular interactions and, in some cases, become overly stable [6,7,8], which may hinder their disassembly after cellular internalization and thereby limit intracellular release and functional availability of cargo molecules. Thus, molecular designs that allow control of assembly stability in response to intracellular environments are needed [9,10]. In this context, mildly acidic pH and reducing conditions have attracted interest as intracellular triggers for modulating peptide assembly stability. pH-responsive control of β-sheet assembly and disassembly has been widely investigated, including in systems designed to respond to mildly acidic conditions relevant to endosomal compartments. In these pH-responsive systems, relatively clear molecular design principles are available: by introducing ionizable or charge-modulating residues into β-sheet-forming peptides, protonation-state changes under acidic conditions can be predictably translated into altered intermolecular interactions, such as increased electrostatic repulsion and/or weakened hydrophobic interactions, thereby promoting disassembly [11,12,13]. In contrast, intracellular reducing environments have been investigated less frequently as a trigger for β-sheet assembly destabilization [14,15,16,17,18]. In previously reported redox-responsive peptide assemblies, disulfide bonds have often been used as structural elements that directly contribute to β-sheet stabilization through intermolecular crosslinking, dimerization, or intramolecular conformational constraint [14,15,16,17]. In these systems, reductive cleavage of the disulfide bonds removes such stabilizing interactions or conformational constraints, thereby promoting destabilization of the β-sheet-rich assemblies. In another interesting approach, coassembly of a short β-sheet-forming peptide with 4,4′-dipyridyl disulfide has been reported to induce structural transformation of amyloid-like assemblies and impart redox-responsive properties to the resulting assemblies [18]. Although these studies demonstrate the usefulness of disulfide chemistry for controlling peptide assemblies, their redox-responsive behavior is closely linked to the specific peptide sequence or peptide/additive combination used in each design. Therefore, it remains unclear how broadly these design concepts can be applied to β-sheet-forming peptide nanofibers as a general strategy for imparting reduction-triggered destabilization.

**Figure 1:**
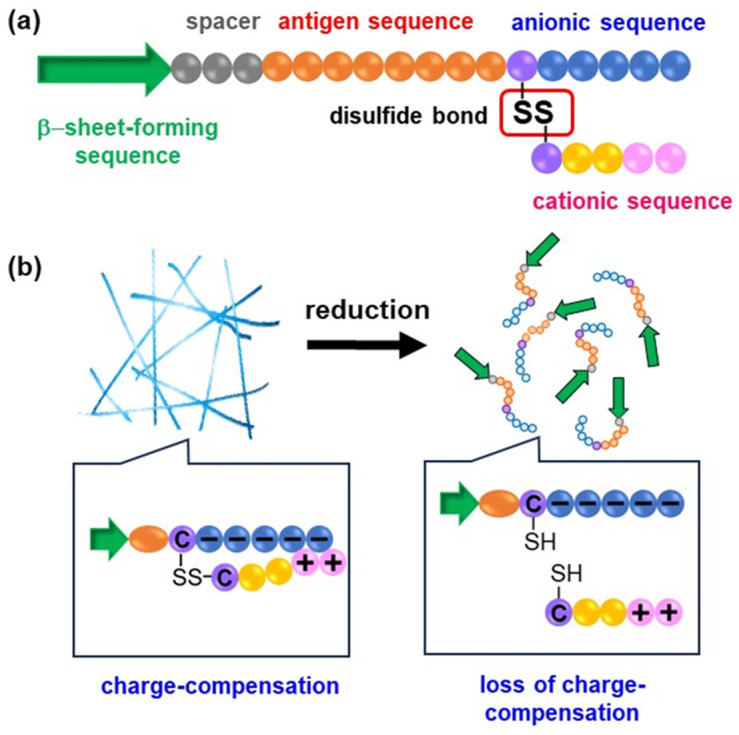
Design concept of ampholytic β-sheet-forming peptides for redox-responsive nanofiber disassembly. (**a**) Schematic illustration of the ampholytic peptide composed of an anionic main-chain peptide (β-sheet-forming motif, spacer, antigenic peptide cargo, cysteine, and oligoglutamic acid segment) and a cationic pendant segment connected through a reducible disulfide linker. (**b**) Schematic illustration of nanofiber disassembly triggered by disulfide bond cleavage under reducing conditions. Cleavage of the disulfide linker releases the cationic pendant segment, enhances electrostatic repulsion among anionic segments, and destabilizes the nanofibers.

In our previous work, we reported antigen-loaded nanofibers formed by the self-assembly of β-sheet-forming peptides (FVIFLD) covalently conjugated to antigenic peptides (SIINFEKL) and hydrophilic chains such as oligo(ethylene glycol) (EG) [19,20]. However, these nanofibers did not include an intracellular trigger-responsive element for disassembly. Building on these studies, we aim to develop β-sheet nanofibers that undergo destabilization and disassembly under reducing conditions. To this end, we propose an ampholytic, charge-compensated design strategy (Figure 1). Unlike these previous approaches, this strategy uses disulfide cleavage to switch the electrostatic balance of the assembling peptide molecules. This charge-balance-based design may provide a transferable framework for imparting reduction-triggered destabilization to existing β-sheet-forming peptide nanofibers.

In this design, the building block molecules consist of an anionic main-chain peptide and a cationic pendant segment linked through a disulfide bond. The main-chain peptide contains a β-sheet-forming motif (FVIFLD), a spacer (GSG), a model antigenic peptide cargo (SIINFEKL), a cysteine residue, and an oligoglutamic acid segment (Figure 1a). The oligoglutamic acid segment is included to suppress nanofiber formation of the main-chain peptide alone under physiological conditions through electrostatic repulsion. To partially offset the negative charges and permit nanofiber formation, a cationic oligopeptide is attached as a pendant segment through the disulfide linkage (Figure 1b). To achieve reduction-triggered disassembly, disulfide cleavage alone is insufficient; it must be coupled to a molecular change that weakens the interactions stabilizing the β-sheet nanofibers. In the present design, disulfide cleavage is expected to release the cationic pendant segment and restore electrostatic repulsion among the anionic main-chain peptides. The restored electrostatic repulsion would then perturb intermolecular interactions and packing between the β-sheet-forming FVIFLD segments, thereby destabilizing the nanofibers. Based on this design rationale, this work systematically investigates a series of designed peptide nanofibers with variations in the relative anionic/cationic charge balance and the position of cationic segment insertion, and evaluates how these structural parameters influence nanofiber formation under physiological conditions and disassembly under reducing conditions.

## 2. Materials and Methods

### 2.1. Materials

4-dimethylaminopyridine (DMAP), *N*, *N*-dimethylformamide (DMF), diethyl ether, hexafluoroisopropanol (HFIP), thioflavin T (ThT), and acetone were purchased from FUJIFILM Wako Pure Chemical Corporation (Osaka, Japan). 2,2′-dipyridyl disulfide was purchased from Tokyo Chemical Industry Co., Ltd. (Tokyo, Japan). Dithiothreitol (DTT) was purchased from Nacalai Tesque, Inc. (Kyoto, Japan). The peptides CHHK-amide, CHHKK-amide, CHHKKK-amide, E_4_, and E_5_ were purchased from GenScript (Piscataway, NJ, USA). The peptides CE_4_, CE_5_, E_4_C, and E_5_C were purchased from Scrum Inc (Tokyo, Japan); their sequences were FVIFLDGSGSIINFEKLCEEEE (CE_4_), FVIFLDGSGSIINFEKLCEEEEE (CE_5_), FVIFLDGSGSIINFEKLEEEEC (E_4_C), and FVIFLDGSGSIINFEKLEEEEEC (E_5_C).

### 2.2. Synthesis

#### 2.2.1. Synthesis of Pyridyl Disulfide-Modified Cationic Peptides

CHHK-amide (40 mg, 77 μmol) and 2,2′-dipyridyl disulfide (67 mg, 306 μmol) were dissolved in degassed DMF (600 μL). DMAP (22 mg, 183 μmol) was added, and the mixture was stirred overnight. HFIP (585 μL) was added to the reaction mixture. The solution was slowly added dropwise to diethyl ether to precipitate the peptide. The resulting precipitate was collected and dried under reduced pressure to afford the product. The product was analyzed by ESI-TOF-MS (micro TOF, Bruker, Billerica, MA, USA): pyridyl-ss-CHHK ([M + 2H]^2+^, *m*/*z* calcd 316.63, found 316.70). The yield was 100%. Pyridyl-ss-CHHKK and pyridyl-ss-CHHKKK were also synthesized by the same procedure and analyzed by ESI-TOF-MS: pyridyl-ss-CHHKK ([M + 2H]^2+^, *m*/*z* calcd 380.68, found 380.67; yield 79%) and pyridyl-ss-CHHKKK ([M + H]^+^, *m*/*z* calcd 888.44, found 888.83; yield 92%).

#### 2.2.2. Synthesis of Ampholytic Peptides

CE_5_ (42 mg, 16 μmol) and pyridyl-ss-CHHK (20 mg, 32 μmol) were dissolved in degassed dry DMF (1.6 mL). DMAP (9.3 mg, 76 μmol) was added, and the mixture was stirred overnight. The reaction solution was slowly added dropwise into acetone to precipitate the peptide. The resulting precipitate was collected and dried under reduced pressure, followed by purification by reversed-phase high-performance liquid chromatography (RP-HPLC) using a Shimadzu system (Shimadzu Corporation, Kyoto, Japan). ESI-TOF-MS analysis confirmed the molecular weight of CE_5_-CHHK ([M + 3H]^3+^, *m*/*z* calcd 1056.83, found 1056.42). Furthermore, E_4_C-CHHK, E_5_C-CHHK, CE_4_-CHHK, CE_5_-CHHKK, and CE_5_-CHHKKK were synthesized and purified by the same procedure and characterized by mass spectrometry. MALDI-TOF-MS (Autoflex Speed, Bruker, Billerica, MA, USA) was employed for E_4_C-CHHK, E_5_C-CHHK, and CE_5_-CHHKKK, whereas ESI-TOF-MS was used for CE_4_-CHHK and CE_5_-CHHKK. The following ion species and *m*/*z* values were obtained: E_4_C-CHHK ([M + H]^+^, *m*/*z* calcd 3040.45, found 3040.50), E_5_C-CHHK ([M + H]^+^, *m*/*z* calcd 3169.48, found 3169.35), CE_4_-CHHK ([M + 3H]^3+^, *m*/*z* calcd 1013.82, found 1013.82), CE_5_-CHHKK ([M + 3H]^3+^, *m*/*z* calcd 1099.53, found 1099.54), and CE_5_-CHHKKK ([M + H]^+^, *m*/*z* calcd 3425.68, found 3425.64).

### 2.3. Preparation of Nanofibers

Each ampholytic peptide was dissolved in dilute aqueous NaOH and then mixed with an equal volume of 8×phosphate-buffered saline (8×PBS) to give a final peptide concentration of 600 μM. The resulting solution was incubated at 37 °C for 21 h. The morphology of the resulting nanofibers was observed by transmission electron microscopy (TEM) using a JEM-2100 microscope (JEOL, Tokyo, Japan) operated at an acceleration voltage of 80 kV. Samples were stained with TI Blue staining solution (Nisshin EM, Tokyo, Japan).

### 2.4. Stability of Nanofibers in PBS

Milli-Q water was added to the nanofiber dispersion prepared in 4×PBS (peptide concentration, 600 μM) at three times the dispersion volume, and time-dependent changes in secondary structure were evaluated by circular dichroism (CD) spectroscopy. CD spectra were recorded at 25 °C on a J-720 spectropolarimeter (JASCO Corporation, Tokyo, Japan). In addition, the morphology of each sample was observed by TEM immediately after adjustment to 1×PBS and after 24 h.

### 2.5. Direct Assembly of Ampholytic Peptides in 1×PBS

Each ampholytic peptide was dissolved in dilute aqueous NaOH and then mixed with an equal volume of 2×PBS to give a final peptide concentration of 600 μM in 1×PBS. The resulting solution was incubated at 37 °C for 21 h, and CD spectra were then recorded on a J-820 spectropolarimeter (JASCO Corporation, Tokyo, Japan). For evaluation using the ThT fluorescence assay, the same procedure was performed in the presence of ThT (20 μM), and fluorescence spectra were recorded using an F-2700 fluorescence spectrophotometer (Hitachi High-Tech Corporation, Tokyo, Japan) with excitation at 450 nm and emission collected from 460 to 600 nm.

### 2.6. Evaluation of Disassembly Behavior of Ampholytic Peptide Nanofibers Under Reducing Conditions

Milli-Q water was added to the nanofiber dispersion prepared in 4×PBS (peptide concentration, 600 μM) at three times the dispersion volume to adjust the dispersion to 1×PBS. The resulting dispersion was incubated at 37 °C for 24 h before the reduction experiment. A concentrated DTT stock solution in PBS was then added to give a final DTT concentration of 2 mM, resulting in a final peptide concentration of 135 μM, and time-dependent changes in secondary structure were evaluated by CD spectroscopy. In addition, the morphology of the samples at 24 h after DTT addition was observed by TEM. As a control, the same evaluations of secondary-structure changes and morphology were performed under conditions in which an equal volume of PBS was added in place of the DTT solution. After DTT treatment, the dispersions were ultracentrifuged at 120,000 rpm for 2 h at 4 °C to separate soluble peptide species from remaining assemblies, and the resulting supernatants were analyzed by RP-HPLC. CE_5_-CHHK was analyzed after 24 h incubation with or without DTT. CE_5_-CHHKKK was analyzed after 6 or 24 h incubation with DTT and after 24 h incubation without DTT. The apparent release ratio was estimated from the amount of CE_5_ main-chain peptide detected in the supernatant relative to the total peptide amount initially used for nanofiber preparation. Peak assignments for CE_5_, intact CE_5_-CHHK, and intact CE_5_-CHHKKK were performed based on mass spectrometric analysis of the corresponding HPLC fractions.

## 3. Results and Discussion

### 3.1. Design and Synthesis of Ampholytic Peptides

To design ampholytic peptides that form nanofibers only upon charge compensation, we selected anionic main-chain peptides containing a β-sheet-forming sequence, a model antigenic peptide sequence, and an oligoglutamic acid segment (Table 1, Figure 1). Preliminary investigation of the self-assembly behavior indicated that the corresponding main-chain peptides E_4_ and E_5_ (Table 1) did not readily maintain fibrillar assemblies under physiologically relevant ionic-strength conditions (Figure S1). These observations indicated that E_4_- and E_5_-based sequences are suitable main-chain components for examining whether the introduction of a cationic segment could induce and stabilize fibrillar assembly through charge compensation. Accordingly, six ampholytic peptides were designed by combining an anionic main-chain peptide with a cationic peptide segment through a reducible disulfide linker (Table 1, Figure 1). As anionic main-chain peptides, four peptides (CE_4_, E_4_C, CE_5_, and E_5_C), differing in the cysteine position and the length of the oligoglutamate segment, were employed. As cationic segments, three peptides (CHHK-amide, CHHKK-amide, and CHHKKK-amide), differing in the number of lysine residues, were used. Ampholytic peptides were synthesized by activating the thiol group of the cysteine residue in the cationic peptide via pyridyl disulfide formation, followed by disulfide bond formation with the cysteine-containing main-chain peptide (Scheme S1).

### 3.2. Self-Assembly and Nanofiber Formation of Ampholytic Peptides

**Figure 2:**
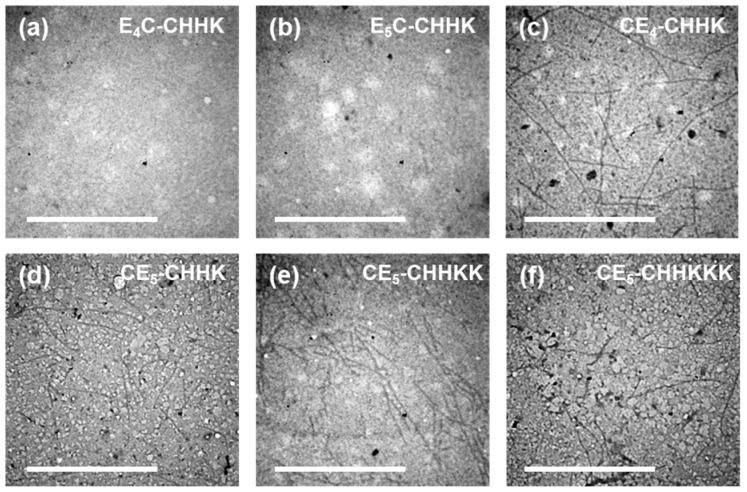
Transmission electron microscopy (TEM) images of ampholytic peptide samples after incubation in 4×phosphate-buffered saline (4×PBS) at 37 °C for 21 h: (**a**) E_4_C-CHHK, (**b**) E_5_C-CHHK, (**c**) CE_4_-CHHK, (**d**) CE_5_-CHHK, (**e**) CE_5_-CHHKK, and (**f**) CE_5_-CHHKKK. Scale bar: 1 μm.

Preliminary experiments using the anionic main-chain peptides E_4_ and E_5_ showed that both peptides could form nanofibers under high-ionic-strength conditions (4×PBS), but these assemblies were not maintained after dilution to PBS (Figure S2). These observations suggested that electrostatic repulsion arising from the anionic segments hindered stable nanofiber formation under physiological ionic-strength conditions, and that additional charge compensation could improve the stability of the assemblies under such conditions. We therefore first examined nanofiber formation of all six ampholytic peptides under 4×PBS and subsequently evaluated the stability of the resulting assemblies after dilution to PBS. Under 4×PBS, nanofiber formation was observed for CE_4_-CHHK, CE_5_-CHHK, CE_5_-CHHKK, and CE_5_-CHHKKK, whereas E_4_C-CHHK and E_5_C-CHHK did not form nanofibers (Figure 2). These results suggest that nanofiber formation is governed not only by the overall anionic/cationic charge balance, but also by the relative placement of the cationic segment with respect to the oligoglutamate segment. In the CE_4_/CE_5_ series, the cationic segment is positioned on the N-terminal side of the oligoglutamate segment, which may allow more effective local compensation of the negative charges of the oligoglutamate segment and thereby reduce electrostatic interference with intermolecular packing of the β-sheet-forming FVIFLD segments. In contrast, in the E_4_C/E_5_C series, positioning of the cationic segment on the C-terminal side of the oligoglutamate segment may be less favorable for compensating the negative charges in a manner that supports nanofiber formation.

### 3.3. Stability of Nanofibers upon Dilution from 4×PBS to PBS

**Figure 3:**
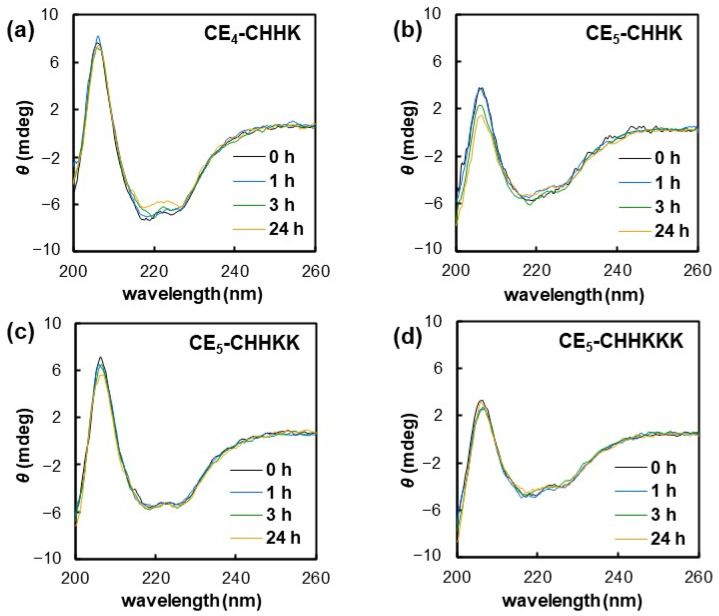
Time-course circular dichroism (CD) spectra of nanofibers after dilution from 4×PBS to 1×PBS, recorded at 0, 1, 3, and 24 h: (**a**) CE_4_-CHHK, (**b**) CE_5_-CHHK, (**c**) CE_5_-CHHKK, and (**d**) CE_5_-CHHKKK.

**Figure 4:**
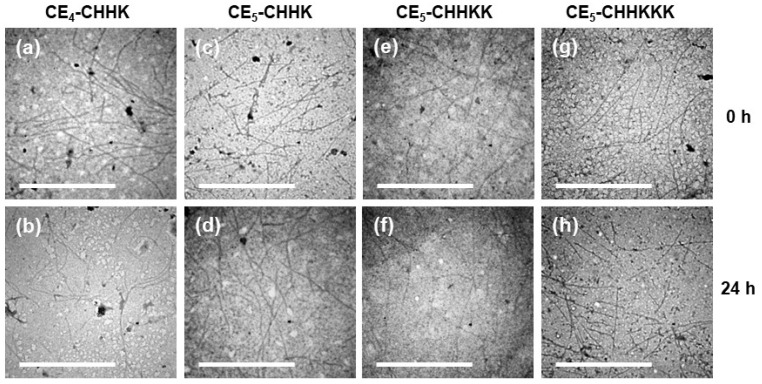
TEM images of nanofiber dispersions at 0 h and 24 h after fourfold dilution from 4×PBS to 1×PBS (*v*/*v* = 1:3) and incubation at 37 °C: (**a**,**b**) CE_4_-CHHK, (**c**,**d**) CE_5_-CHHK, (**e**,**f**) CE_5_-CHHKK, and (**g**,**h**) CE_5_-CHHKKK. Images were taken immediately after dilution (0 h; (**a**,**c**,**e**,**g**)) and after 24 h (**b**,**d**,**f**,**h**). Scale bar: 1 μm.

To test whether the nanofibers formed by the four ampholytic peptides in 4×PBS (CE_4_-CHHK, CE_5_-CHHK, CE_5_-CHHKK, and CE_5_-CHHKKK) persist under physiological ionic strength, we diluted the dispersions fourfold with water (*v*/*v* = 1:3) to 1×PBS and monitored time-dependent changes in secondary structure by CD spectroscopy (Figure 3). Immediately after dilution to 1×PBS, all four preformed nanofiber samples exhibited a negative Cotton band around 220 nm, characteristic of β-sheet structures [21], and no marked changes in peak intensity or spectral shape were observed over the subsequent 24 h. Consistent with the CD results, TEM confirmed abundant nanofibers in all samples immediately after dilution and after 24 h (Figure 4). Collectively, these results indicate that nanofibers preformed in 4×PBS retained β-sheet-rich secondary structures and fibrillar morphologies for at least 24 h after dilution to 1×PBS.

To examine whether the ampholytic peptides can assemble directly in 1×PBS without pre-assembly in 4×PBS, we further investigated the assembly behavior of the four peptides (CE_4_-CHHK, CE_5_-CHHK, CE_5_-CHHKK, and CE_5_-CHHKKK). The assembly behavior was evaluated by the ThT fluorescence assay and CD spectroscopy. The ThT fluorescence measured in the presence of each peptide after incubation in 1×PBS was markedly higher than that of ThT alone (Figure S3). The CD spectra of the samples incubated directly in 1×PBS (Figure S4) were similar to those obtained after dilution of the nanofibers preformed in 4×PBS to 1×PBS (Figure 3). These results suggest that these ampholytic peptides can form β-sheet-rich assemblies directly in 1×PBS, and that the assemblies retained after dilution from 4×PBS to 1×PBS cannot be explained solely as kinetically trapped structures formed under high-ionic-strength conditions.

### 3.4. Disulfide Cleavage–Dependent Destabilization and Disassembly Behavior

**Figure 5:**
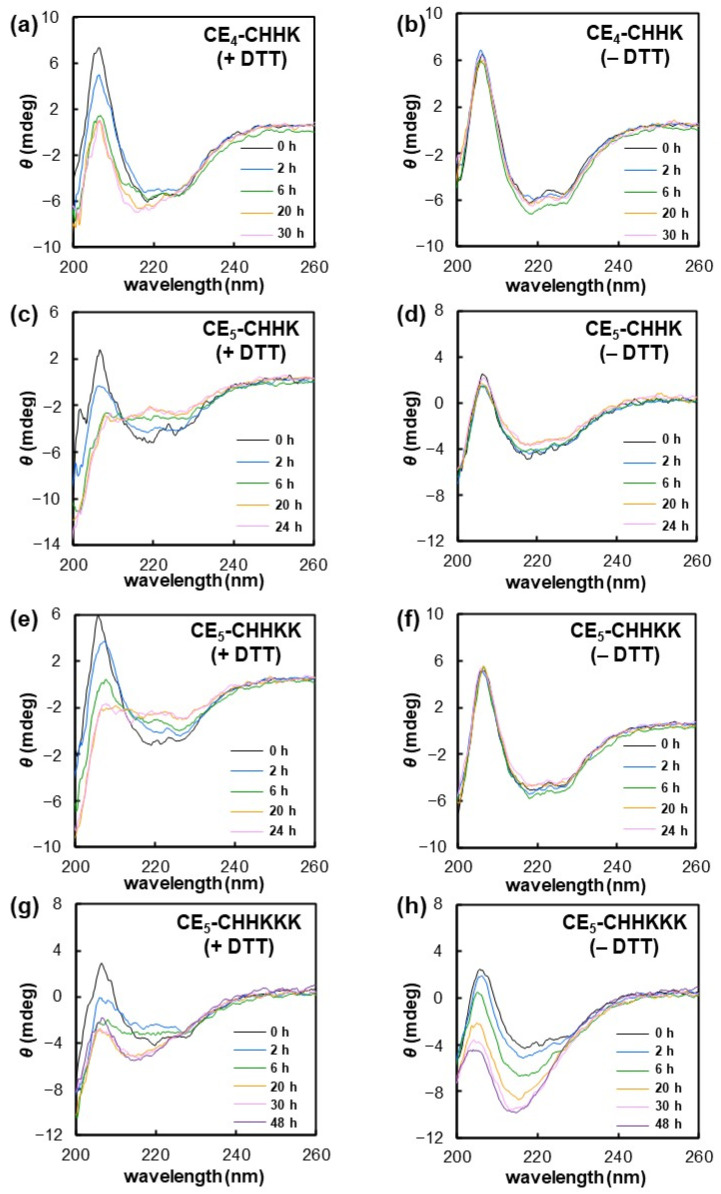
Time-course CD spectra of nanofibers after addition of dithiothreitol (DTT): (**a**,**b**) CE_4_-CHHK, (**c**,**d**) CE_5_-CHHK, (**e**,**f**) CE_5_-CHHKK, and (**g**,**h**) CE_5_-CHHKKK. A DTT solution in PBS was added to the samples (**a**,**c**,**e**,**g**), whereas the same volume of PBS without DTT was added to the corresponding control samples (**b**,**d**,**f**,**h**). In both conditions, the final peptide concentration was 135 μM.

**Figure 6:**
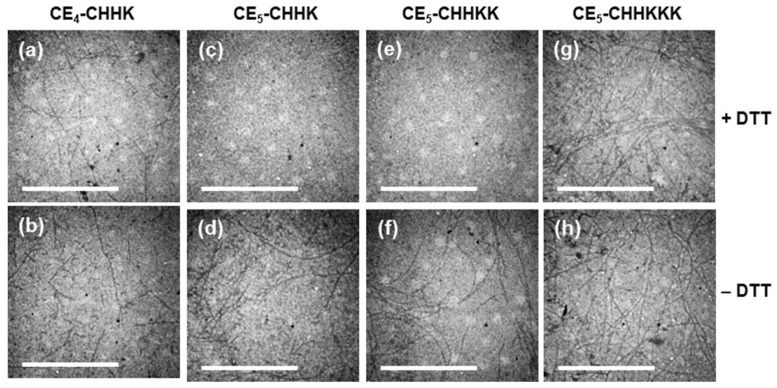
TEM images of nanofibers 24 h after addition of DTT: (**a**,**b**) CE_4_-CHHK, (**c**,**d**) CE_5_-CHHK, (**e**,**f**) CE_5_-CHHKK, and (**g**,**h**) CE_5_-CHHKKK. A DTT solution in PBS was added to the samples (**a**,**c**,**e**,**g**), whereas the same volume of PBS without DTT was added to the corresponding control samples (**b**,**d**,**f**,**h**). Scale bar: 1 μm.

Next, redox-induced secondary-structure changes in the four ampholytic peptide nanofibers (CE_4_-CHHK, CE_5_-CHHK, CE_5_-CHHKK, and CE_5_-CHHKKK) were examined (Figure 5). The nanofiber dispersions were first prepared in 4×PBS, diluted to 1×PBS, and incubated at 37 °C for 24 h. A DTT stock solution in PBS was then added to the nanofiber dispersions to give a final DTT concentration of 2 mM and a final peptide concentration of 135 μM, and the time-dependent secondary-structure changes after DTT addition were monitored by CD spectroscopy. The corresponding morphological changes were evaluated by TEM (Figure 6). Although DTT treatment does not directly reproduce physiological intracellular reducing conditions, it was employed as a strong reductant for initial experimental validation of the design concept that reduction in the disulfide linkage can induce nanofiber destabilization through loss of charge compensation. As a control, the same volume of PBS without DTT was added, and the samples were analyzed under otherwise identical conditions. Overall, the four nanofibers showed three distinct secondary-structure responses under reducing conditions: (i) little change in β-sheet-rich structure (CE_4_-CHHK), (ii) pronounced β-sheet loss (CE_5_-CHHK and CE_5_-CHHKK), and (iii) transient β-sheet loss followed by restabilization of a β-sheet-rich structure (CE_5_-CHHKKK).

For CE_4_-CHHK nanofibers, the negative Cotton band around 220 nm was maintained up to 30 h after DTT addition (Figure 5a). In the absence of DTT, no appreciable spectral change was observed over the same period (Figure 5b). TEM observations confirmed nanofibers both with and without DTT, consistent with the CD results (Figure 6a,b). These results indicate that CE_4_-CHHK nanofibers retain a β-sheet-rich structure and fibrillar morphology even under reducing conditions.

For CE_5_-CHHK and CE_5_-CHHKK, a negative Cotton effect at approximately 220 nm was observed immediately after DTT addition. Under reducing conditions, the magnitude of the negative Cotton band at approximately 220 nm gradually diminished over time, and a negative Cotton band near 200 nm, characteristic of a random-coil structure [22], became more prominent, suggesting a conformational transition from a β-sheet to a random-coil structure (Figure 5c,e). In contrast, in the absence of DTT, the negative Cotton band characteristic of the β-sheet structure at ~220 nm was maintained for at least 24 h (Figure 5d,f). Consistent with the CD results, TEM observations at 24 h showed that intact nanofibers were far less abundant under DTT-treated conditions (Figure 6c,e) than in the corresponding samples without DTT (Figure 6d,f). Collectively, these findings suggest that nanofibers formed by CE_5_-CHHK and CE_5_-CHHKK undergo a β-sheet-to-random-coil transition accompanied by disassembly under reducing conditions. This behavior is consistent with disulfide bond cleavage–induced release of the cationic peptide segment, leading to loss of charge compensation and destabilization of the nanofibers.

For CE_5_-CHHKKK, in the presence of DTT, the negative Cotton band near 220 nm decreased in magnitude during the first 2 h after DTT addition and then gradually increased again, accompanied by a shift toward shorter wavelengths, until 20 h after DTT addition (Figure 5g). The early spectral changes (≤2 h) resembled those observed for CE_5_-CHHK and CE_5_-CHHKK and were consistent with transient destabilization of the β-sheet structure upon disulfide bond cleavage. The subsequent recovery of the β-sheet-associated signature may be attributable to the longer cationic peptide segment in CE_5_-CHHKKK, which could retain electrostatic interactions with the anionic main-chain peptide even after loss of covalent tethering. TEM observations at 24 h confirmed the presence of nanofibers under reducing conditions (Figure 6g), consistent with re-stabilization after transient destabilization. In the absence of DTT, the magnitude of the negative Cotton band near 220 nm increased over time while gradually shifting toward shorter wavelengths (Figure 5h), and TEM observations at 24 h confirmed the presence of nanofibers (Figure 6h). Because the overall CD spectral profile remained almost unchanged during the first 24 h after dilution from 4×PBS to 1×PBS (Figure 3), the spectral changes observed in the DTT-free control are unlikely to be caused by the initial dilution itself. Rather, they may reflect structural maturation or reorganization of the preformed nanofibers triggered by the slight dilution caused by the addition of PBS during preparation of the DTT-free control. This behavior may be related to the fact that CE_5_-CHHKKK contains the largest and most highly cationic pendant segment among the peptides examined. Such a bulky and charge-rich pendant segment may affect molecular packing in the nanofiber core containing the β-sheet-forming FVIFLD segment, making the preformed nanofibers more susceptible to structural reorganization upon slight dilution. Overall, these results suggest that CE_5_-CHHKKK undergoes transient destabilization followed by re-stabilization rather than complete disassembly under reducing conditions.

To obtain quantitative information on the reduction-induced release of peptide components from the nanofibers, the supernatants of CE_5_-CHHK and CE_5_-CHHKKK nanofiber dispersions after ultracentrifugation were analyzed by RP-HPLC (Figure S5). For CE_5_-CHHK, intact CE_5_-CHHK corresponding to approximately 10 mol% of the total peptide used for nanofiber preparation was detected in the supernatant under the DTT-free control condition, whereas the CE_5_ main-chain peptide corresponding to approximately 29 mol% of the total peptide was detected after 24 h of DTT treatment. For CE_5_-CHHKKK, intact CE_5_-CHHKKK corresponding to approximately 4 mol% of the total peptide was detected in the DTT-free control, whereas the CE_5_ main-chain peptide corresponding to approximately 36 mol% of the total peptide was detected after both 6 and 24 h of DTT treatment. The detected CE_5_ main-chain peptide corresponds to the antigen-containing anionic peptide generated by disulfide cleavage. For both CE_5_-CHHK and CE_5_-CHHKKK, the amount of CE_5_ main-chain peptide detected in the supernatant after DTT treatment exceeded the amount of intact peptide detected in the corresponding DTT-free control. These results indicate that DTT treatment cleaved the disulfide linkages in the peptides within the nanofibers, resulting in the release of the antigen-containing CE_5_ main-chain peptide from the nanofibers. Notably, for CE_5_-CHHKKK, the comparable amounts of CE_5_ main-chain peptide detected after 6 and 24 h of DTT treatment suggest that the structural recovery observed by CD spectroscopy does not necessarily indicate reassociation of the released CE_5_ peptide into nanofibers, but may instead reflect structural changes in the peptide components remaining in the nanofibers. It should be noted that these values were calculated from the peptide species detected in the supernatant after ultracentrifugation and therefore represent apparent release ratios, rather than the total amount of peptide components dissociated from the nanofibers.

Collectively, these results indicate that the responses of the nanofibers to reducing conditions, including reduction-insensitive stability, disassembly, and transient destabilization followed by re-stabilization, are largely governed by peptide charge design, particularly the relative electrostatic balance between the anionic main-chain peptide and the cationic segment. Redox-triggered destabilization appears to require sufficient anionic character in the main-chain peptide, such that the main-chain peptide alone does not stably form nanofibers under physiological ionic-strength conditions. If this anionic character is insufficient, the assemblies can remain stable even after disulfide cleavage. At the same time, the cationic segment must be able to support nanofiber formation before reduction by compensating electrostatic repulsion among the anionic main-chain peptides. However, an excessively long or highly cationic segment may retain electrostatic interactions with the anionic main-chain peptide even after disulfide cleavage, thereby causing post-cleavage re-stabilization rather than sustained disassembly. Thus, an appropriate cationic segment should have a moderate length and charge that balance these two requirements: supporting nanofiber formation before reduction while avoiding re-stabilization after cleavage. When this strategy is applied to different antigenic cargos, the anionic residue number in the main-chain peptide and the length/charge of the cationic segment may need to be adjusted based on the design insights obtained in this study, while taking into account the charge and hydrophobicity of the cargo.

## 4. Conclusions

In this study, we demonstrated that charge-compensated ampholytic design can be used to tune redox-responsive destabilization of β-sheet peptide nanofibers. Four of the designed ampholytic peptides, comprising an anionic β-sheet-forming main-chain and a disulfide-linked cationic segment, formed nanofibers under high ionic strength (4×PBS), and the preformed nanofibers retained β-sheet-rich secondary structures and fibrillar morphologies for at least 24 h after dilution from 4×PBS to 1×PBS. Under reducing conditions, disassembly was observed for CE_5_-CHHK and CE_5_-CHHKK, whereas CE_4_-CHHK and CE_5_-CHHKKK retained fibrillar structures; notably, CE_5_-CHHKKK exhibited transient destabilization followed by re-stabilization. These results suggest that post-reduction nanofiber stability and disassembly behavior are governed by the relative electrostatic balance between the anionic main-chain peptide and the cationic segment. More specifically, redox-triggered disassembly appears to require sufficient anionic character in the main-chain peptide and a cationic segment that is not excessively long. Overall, this work provides a molecular design strategy for tuning the redox-responsive destabilization of β-sheet peptide nanofibers by exploiting reduction-triggered loss of charge compensation. Further studies using biologically relevant cell-free conditions are needed to gain insight into how these nanofibers may respond to intracellular environments, including endosome-relevant acidic pH and glutathione-containing reducing conditions. Subsequent cellular experiments will also be important to verify whether such redox-responsive destabilization behavior enhances intracellular cargo release and functional activity.

## Figures and Tables

**Table 1:** Peptide sequence used in this study.

Abbreviation	Sequence of Peptides
E_4_	**FVIFLDGSGSIINFEKL**-**EEEE**
E_5_	**FVIFLDGSGSIINFEKL**-**EEEEE**
E_4_C-CHHK	**FVIFLDGSGSIINFEKL**-**EEEEC**(-ss-**CHHK**-amide)
E_5_C-CHHK	**FVIFLDGSGSIINFEKL**-**EEEEEC**(-ss-**CHHK**-amide)
CE_4_-CHHK	**FVIFLDGSGSIINFEKL**-**C**(-ss-**CHHK**-amide)**EEEE**
CE_5_-CHHK	**FVIFLDGSGSIINFEKL**-**C**(-ss-**CHHK**-amide)**EEEEE**
CE_5_-CHHKK	**FVIFLDGSGSIINFEKL**-**C**(-ss-**CHHKK**-amide)**EEEEE**
CE_5_-CHHKKK	**FVIFLDGSGSIINFEKL**-**C**(-ss-**CHHKKK**-amide)**EEEEE**

The β-sheet-forming sequence, spacer, antigen epitope, Cys, His, Lys, and Glu are shown in green, gray, orange, purple, yellow, pink, and blue, respectively. The abbreviation “ss” denotes a disulfide bond, and the C-termini of the cationic segments are amidated.

## Data Availability

The raw data supporting the conclusions of this article will be made available by the authors on request.

## Supplementary Materials

The following supporting information can be downloaded at: https://www.mdpi.com/article/10.3390/polym18111291/s1, Scheme S1: Synthetic schemes for (a) pyridyl disulfide-modified cationic peptides and (b) ampholytic peptides; Figure S1: TEM images of E_4_ and E_5_ samples after incubation in 5 mM McIlvaine buffer (pH 7.4) containing 150 mM NaCl: (a) E4 and (b) E5. Scale bar: 1 μm; Figure S2: TEM images of E_4_ and E_5_ samples after incubation in 4×PBS and after subsequent dilution to PBS: (a,b) E4 and (c,d) E5. Samples after incubation in 4×PBS are shown in (a, c), whereas the corresponding samples 24 h after fourfold dilution into PBS are shown in (b,d). Scale bar: 1 μm; Figure S3: ThT fluorescence spectra recorded after incubation in PBS for 24 h with or without ampholytic peptides. Ampholytic peptides were incubated directly in PBS with ThT for 24 h without the 4×PBS pre-assembly step, and fluorescence spectra were then recorded. Dashed lines indicate ThT alone, and solid lines indicate ThT in the presence of peptide. (a) CE4-CHHK, (b) CE5-CHHK, (c) CE5-CHHKK, and (d) CE5-CHHKKK; Figure S4: CD spectra of ampholytic peptide assemblies formed in PBS. The four ampholytic peptides were incubated directly in PBS for 24 h without the 4×PBS pre-assembly step, and their secondary structures were evaluated by CD spectroscopy. (a) CE4-CHHK, (b) CE5-CHHK, (c) CE5-CHHKK, and (d) CE5-CHHKKK; Figure S5: RP-HPLC analysis of soluble peptide species generated from CE_5_-CHHK and CE_5_-CHHKKK nanofibers after DTT treatment. Supernatants obtained after ultracentrifugation of nanofiber dispersions were analyzed by RP-HPLC to evaluate the release of soluble CE5 main-chain peptide species. (a) RP-HPLC chromatograms of CE5-CHHK nanofibers after 24 h incubation with DTT (top) or without DTT (bottom). (b) RP-HPLC chromatograms of CE5-CHHKKK nanofibers after 6 h incubation with DTT (top), 24 h incubation with DTT (middle), or 24 h incubation without DTT (bottom). Peaks corresponding to CE5, intact CE5-CHHK, and intact CE5-CHHKKK were assigned based on mass spectrometric analysis of the corresponding HPLC fractions.
