# Supplementary material for: Charge-Balanced Design for Redox-Responsive Disassembly of Ampholytic β-Sheet Peptide Nanofibers
Source: Polymers (Basel). 2026 May 24;18(11):1291. doi: 10.3390/polym18111291 (PMC13259212; doi:10.3390/polym18111291)
Supplement: Supplementary file 1 [file polymers-18-01291-s001.zip › polymers-4259682-supplementary.pdf]

Supporting Information

Charge-Balanced Design for Redox-Responsive Disassembly of Ampholytic β-Sheet Peptide Nanofibers

Tomonori Waku [1,2,*], Kaede Akita [1], Takehiro Deromachi [1], Kazuya Matsuo [1] and Akio Kobori [1]

[1] Faculty of Molecular Chemistry and Engineering, Kyoto Institute of Technology, Matsugasaki, Sakyo-ku, Kyoto 606-8585, Japan;
[2] Center for Social and Biomedical Engineering, Kyoto Institute of Technology, Matsugasaki, Sakyo-ku,
Kyoto 606-8585, Japan
* Correspondence: waku1214@kit.ac.jp; Tel.: +81-75-724-7811

Contents

Scheme S1. Synthetic schemes for (a) pyridyl disulfide-modified cationic peptides and (b) ampholytic peptides.

Figure S1. TEM images of E_4 and E_5 samples after incubation in 5 mM McIlvaine buffer (pH 7.4) containing 150 mM NaCl: (a) E_4 and (b) E_5. Scale bar: 1 μm.

Figure S2. TEM images of E_4 and E_5 samples after incubation in 4×PBS and after subsequent dilution to PBS: (a, b) E_4 and (c, d) E_5. Samples after incubation in 4×PBS are shown in (a, c), whereas the corresponding samples 24 h after fourfold dilution into PBS are shown in (b, d). Scale bar: 1 μm.

Figure S3. ThT fluorescence spectra recorded after incubation in PBS for 24 h with or without ampholytic peptides. Ampholytic peptides were incubated directly in PBS with ThT for 24 h without the 4×PBS pre-assembly step, and fluorescence spectra were then recorded. Dashed lines indicate ThT alone, and solid lines indicate ThT in the presence of peptide. (a) CE_4-CHHK, (b) CE_5-CHHK, (c) CE_5-CHHKK, and (d) CE_5-CHHKKK.

Figure S4. CD spectra of ampholytic peptide assemblies formed in PBS. The four ampholytic peptides were incubated directly in PBS for 24 h without the 4×PBS pre-assembly step, and their secondary structures were evaluated by CD spectroscopy. (a) CE_4-CHHK, (b) CE_5-CHHK, (c) CE_5-CHHKK, and (d) CE_5-CHHKKK.

Figure S5. RP-HPLC analysis of soluble peptide species generated from CE_5-CHHK and CE_5-CHHKKK nanofibers after DTT treatment. Supernatants obtained after ultracentrifugation of nanofiber dispersions were analyzed by RP-HPLC to evaluate the release of soluble CE5 main-chain peptide species. (a) RP-HPLC chromatograms of CE_5-CHHK nanofibers after 24 h incubation with DTT (top) or without DTT (bottom). (b) RP-HPLC chromatograms of CE_5-CHHKKK nanofibers after 6 h incubation with DTT (top), 24 h incubation with DTT (middle), or 24 h incubation without DTT (bottom). Peaks corresponding to CE_5, intact CE_5-CHHK, and intact CE_5-CHHKKK were assigned based on mass spectrometric analysis of the corresponding HPLC fractions.

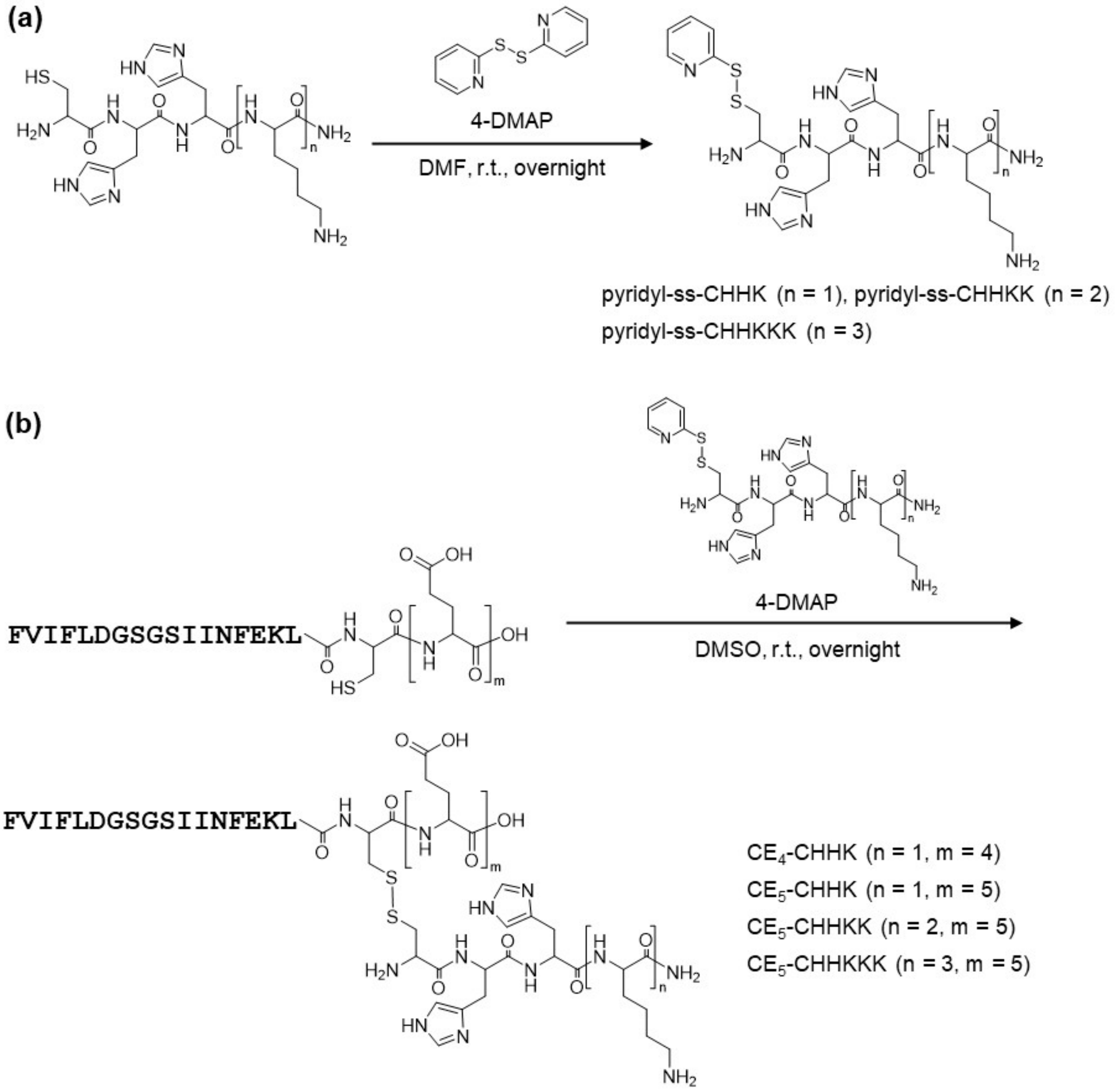

Scheme S1. Synthetic schemes for (a) pyridyl disulfide-modified cationic peptides and (b) ampholytic peptides.

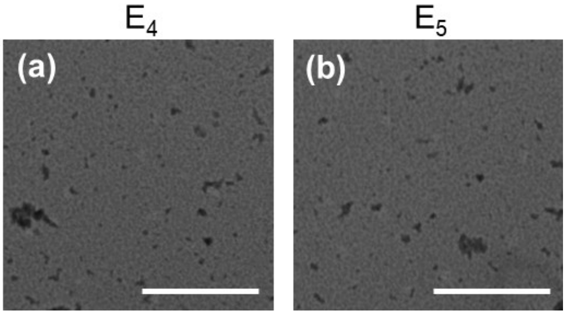

Figure S1. TEM images of E_4 and E_5 samples after incubation in 5 mM McIlvaine buffer (pH 7.4) containing 150 mM NaCl: (a) E_4 and (b) E_5. Scale bar: 1 μm.

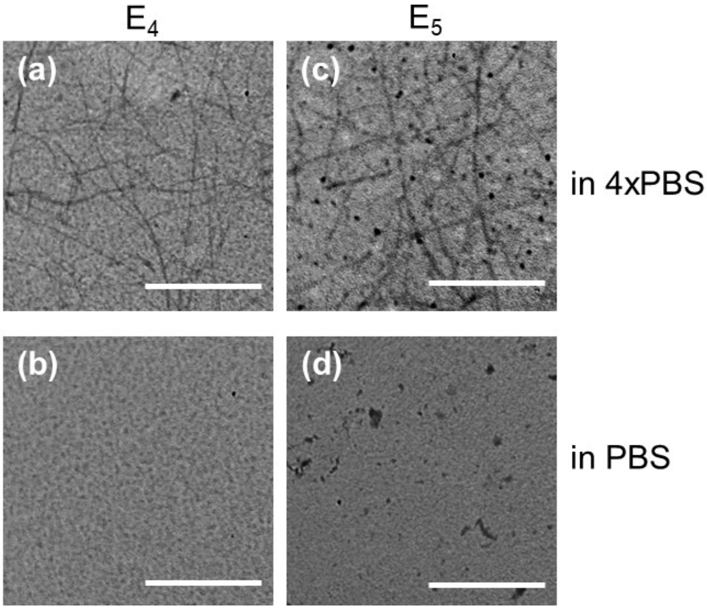

Figure S2. TEM images of E_4 and E_5 samples after incubation in 4×PBS and after subsequent dilution to PBS: (a, b) E_4 and (c, d) E_5. Samples after incubation in 4×PBS are shown in (a, c), whereas the corresponding samples 24 h after fourfold dilution into PBS are shown in (b, d).

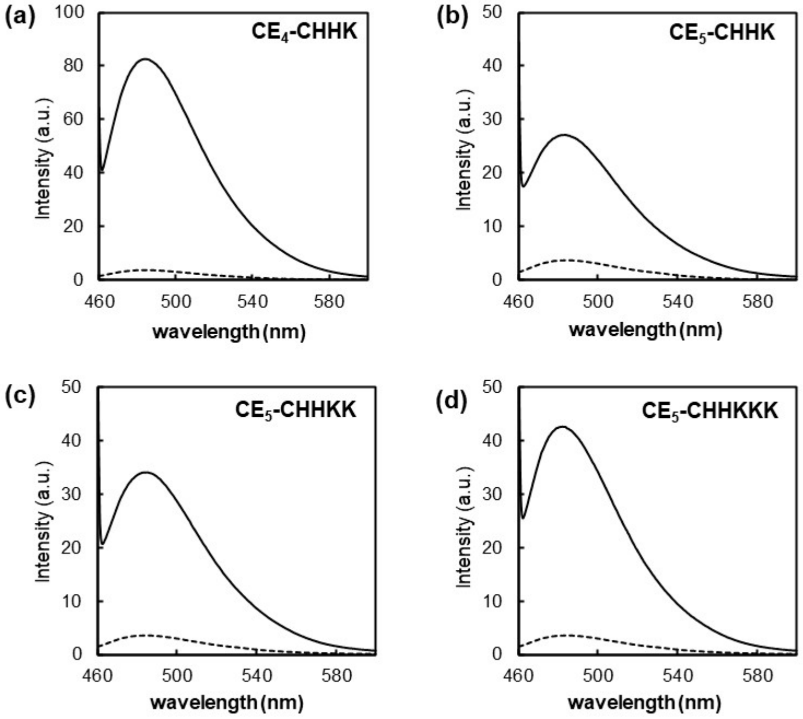

Figure S3. ThT fluorescence spectra recorded after incubation in PBS for 24 h with or without ampholytic peptides. Ampholytic peptides were incubated directly in PBS with ThT for 24 h without the 4×PBS pre-assembly step, and fluorescence spectra were then recorded. Dashed lines indicate ThT alone, and solid lines indicate ThT in the presence of peptide. (a) CE_4-CHHK, (b) CE_5-CHHK, (c) CE_5-CHHKK, and (d) CE_5-CHHKKK.

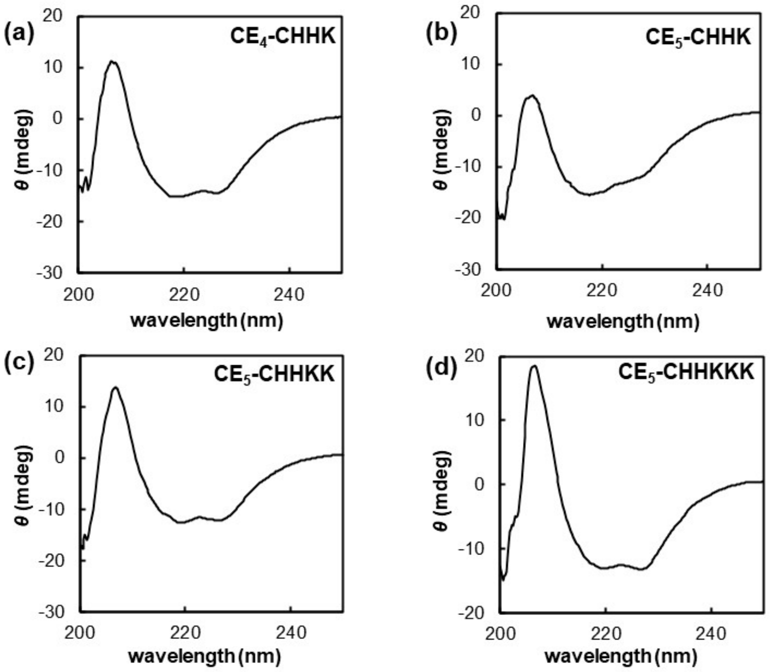

Figure S4. CD spectra of ampholytic peptide assemblies formed in PBS. The four ampholytic peptides were incubated directly in PBS for 24 h without the 4×PBS pre-assembly step, and their secondary structures were evaluated by CD spectroscopy. (a) CE_4-CHHK, (b) CE_5-CHHK, (c) CE_5-CHHKK, and (d) CE_5-CHHKKK.

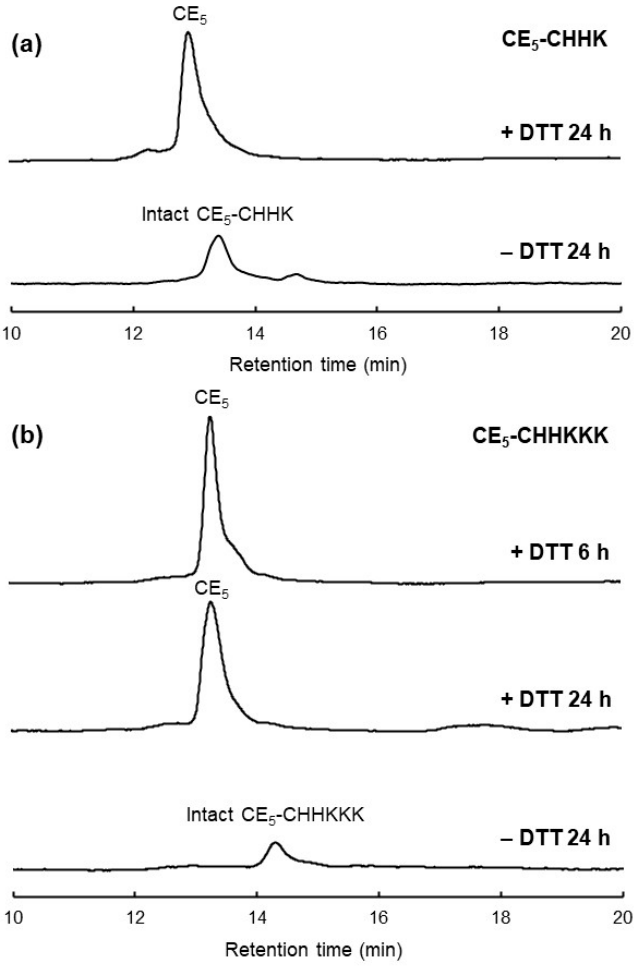

Figure S5. RP-HPLC analysis of soluble peptide species generated from CE_5-CHHK and CE_5-CHHKKK nanofibers after DTT treatment. Supernatants obtained after ultracentrifugation of nanofiber dispersions were analyzed by RP-HPLC to evaluate the release of soluble CE5 main-chain peptide species. (a) RP-HPLC chromatograms of CE_5-CHHK nanofibers after 24 h incubation with DTT (top) or without DTT (bottom). (b) RP-HPLC chromatograms of CE_5-CHHKKK nanofibers after 6 h incubation with DTT (top), 24 h incubation with DTT (middle), or 24 h incubation without DTT (bottom). Peaks corresponding to CE_5, intact CE_5-CHHK, and intact CE_5-CHHKKK were assigned based on mass spectrometric analysis of the corresponding HPLC fractions.
